# Supplementary material for: Determinants of Refugee and Migrant Health Status in 10 European Countries: The Mig-HealthCare Project
Source: Int J Environ Res Public Health. 2020 Aug 31;17(17):6353. doi: 10.3390/ijerph17176353 (PMC7503735; doi:10.3390/ijerph17176353)
Supplement: Supplementary file 1 [file ijerph-17-06353-s001.pdf]

Supplementary Materials

**Table 1.** Characteristics of the total migrants and refugees (N=1407).	
	N (%)
Country of Interview	
Austria	126 (8.96)
Bulgaria	226 (16.06)
Cyprus	110 (7.82)
France	64 (4.55)
Germany	11 (0.78)
Greece	255 (18.12)
Italy	271 (19.26)
Malta	38 (2.7)
Spain	202 (14.36)
Sweden	104 (7.39)
Five most frequent countries of origin	
Syria	294 (21.15)
Afghanistan	211 (15.18)
Iraq	127 (9.14)
Nigeria	118 (8.49)
Iran	48 (3.45)
Males	889 (63.18)
Final Destination (Yes)	979 (72.73)
Asylum (Yes)	358 (28.94)
Other kind of permission (Yes)	619 (46.37)
	Mean ± Standard deviation
Age (years)	31.98±11.05
BMI (kg/m^2)	24.79±4.42
Education (years)	9.1±5

Table 2. Characteristics of migrants and refugees by country of interview.

	Austria (N=126)	Bulgaria (N=226)	Cyprus (N=110)	France (N=64)	Germany (N=11)	Greece (N=255)	Italy (N=271)	Malta (N=38)	Spain (N=202)	Sweden (N=104)
Country of origin	31(24.	74(32.7	26(23.6	2(3.13)	9(81.82	105(41.	2(0.74)	2(5.26)	2(0.99)	41(39.4

(%)<br>Syria<br>Afghanistan<br>Iraq<br>Nigeria<br>Iran<br>Other	60)<br>10 (7.94)<br>26(20.63)<br>9(7.14)<br>6(4.76)<br>44 (34.92)	4)<br>74(32.74)<br>36(15.93)<br>1(0.44)<br>21(9.29)<br>20 (8.85)	4)<br>0(0)<br>21(19.09)<br>4(3.64)<br>0(0)<br>59(53.64)	22(34.38)<br>0(0)<br>0(0)<br>0(0)<br>40 (62.50)	)<br>0(0)<br>0(0)<br>0(0)<br>0(0)<br>2 (18.18)	18)<br>91(35.69)<br>32(12.55)<br>0(0)<br>17(6.67)<br>10(3.92)	1(0.37)<br>0(0)<br>97(35.79)<br>0(0)<br>171(63.10)	2(5.26)<br>5(13.16)<br>3(7.89)<br>0(0)<br>26(68.42)	2(0.99)<br>0(0)<br>1(0.5)<br>0(0)<br>197(97.52)	2)<br>9(8.65)<br>7(6.73)<br>3(2.88)<br>4(3.85)<br>40(38.46)
Males (%)	63 (50)	142 (62.83)	65 (59.09)	45 (70.31)	3 (27.27)	149 (58.43)	236 (87.08)	25 (65.79)	114 (56.44)	47 (45.19)
Final Destination (%Yes)	92 (74.19)	138 (61.61)	74 (68.52)	44 (91.67)	11 (100)	84 (36.21)	246 (93.18)	16 (51.61)	180 (89.11)	94 (92.16)
Asylum (%Yes)	54 (43.20)	30 (23.26)	21 (20.39)	27 (65.85)	3 (27.27)	85 (37.61)	41 (15.47)	8 (23.53)	47 (23.62)	42 (40.38)
Other permission (%Yes)	65 (52.85)	1 (0.44)	56 (54.90)	19 (43.18)	8 (80)	101 (44.69)	211 (79.32)	30 (90.91)	72 (35.82)	56 (53.85)
Age (years)	31.63±9.82	31.57±12.01	34.20±10.43	28.92±7.72	44.64±16.64	34.88±10.20	25.27±5.76	34.11±11.08	35.38±12.86	35.32±11.43
BMI (kg/m^2)	24.42±4.11	24.79±4.62	26.29±4.10	24.90±5.83	26.53±5.21	25.03±5.20	23.79±2.98	24.40±3.77	24.90±4.10	25.39±5.30

**Table 3.** Health status of migrants and refugees.	
	N (%)
Headaches / Migraines	179 (12.72)
Presence of mental health problem	108 (7.68)
Sleep disorders	95 (6.75)
Illness related to bone and muscle	89 (6.33)
Gastrointestinal disease	82 (5.83)
Respiratory disease	72 (5.12)
Diabetes	59 (4.19)
Hypertension	59 (4.19)
Chronic problems from Injury/accidents	51 (3.63)
Heart disease	44 (3.13)
Kidney disease	35 (2.49)
Tuberculosis	9 (0.64)
Cancer	6 (0.43)
AIDS/HIV	5 (0.36)
Brain stroke	4 (0.29)
General Health score (mean±sd)	63.6±23.4

**Table 4.** Access and interaction with health care services.	
	N (%)

Inability to access health care services during the last 6 months.	603 (45.2)
Need for a translator during their interactions with healthcare services:	
Never	348 (26.7)
Few times	237 (18.2)
Most times	253 (19.4)
Always	464 (35.6)
Did you need to take medication and were not able to?	
Not needed	430 (38.5)
Needed and had access	541 (48.5)
Needed and was not able to take it	145 (13.0)
Do you believe that you have worse access to health services compared to local people? (Yes)	322 (26.7)

Table 5. Multivariable linear regression models regarding physical and mental health status, country of interview, and country of origin in migrants and refugees with general health as a dependent variable.

Model 1 *(N=1129)*	Estimates	*P-value*	95% Confidence Interval
Presence of mental health problem	-15.45	**<0.001**	(-20.28 , -10.63)
Heart disease	-15.13	**<0.001**	(-22.89 , -7.37)
Illness related to bone and muscle	-11.24	**<0.001**	(-16.63 , -5.86)
Hypertension	-11.91	**<0.001**	(-18.89 , -4.94)
Gastrointestinal disease	-7.59	**<0.001**	(-12.78 , -2.41)
Respiratory disease	-8.35	**0.003**	(-13.85 , -2.84)
Cancer	-24.57	**0.006**	(-42.04 , -7.11)
Tuberculosis	-20.6	**0.005**	(-34.8 , -6.41)
Kidney disease	-8.87	**0.041**	(-17.39 , -0.35)
Model 2 *(N=1129)*			
Presence of mental health problem	-14.31	**<0.001**	(-19.39 , -9.23)
Sleep disorders	0.54	0.841	(-4.78 , 5.87)
Headaches / Migraines	-1.97	0.324	(-5.88 , 1.94)
Having one disease or chronic condition	-2.74	0.085	(-5.86 , 0.38)
Having at least two diseases or chronic conditions	-16.65	**<0.001**	(-20.4 , -12.9)
Model 3 *(N=1054)*			
Germany or Austria as country of interview+	8.66	**0.002**	(3.21 , 14.11)
Bulgaria as country of interview+	-6.94	**0.028**	(-13.13 , -0.74)
Spain as country of interview+	9.84	**<0.001**	(5.04 , 14.65)
Afghanistan as country of origin+	-5.42	**0.032**	(-10.37 , -0.47)
Iraq as country of origin+	-7.76	**<0.001**	(-12.14 , -3.39)
Nigeria as country of origin+	-2.38	0.393	(-7.83 , 3.08)
Other country of origin+	-5.4	0.095	(-11.76 , 0.95)
Having other kind of permission	3.23	**0.034**	(0.24 , 6.21)
Having one disease or chronic condition	-4.94	**0.003**	(-8.18 , -1.71)
Having at least two diseases or chronic conditions	-18.15	**<0.001**	(-21.36 , -14.93)

+compared with Italy as country of interview and with Syria as country of origin

All models are adjusted for age and sex. Model 1 is adjusted for diabetes, brain stroke and AIDS/HIV. Model 3 is adjusted for final destination (yes/no), other countries of interview and having asylum (yes/no).

Table 6. Pearson's correlation for each question with total general health score.

**18**	In general, would you say your health is: good etc.
**18a**	(a) I seem to get sick a little easier than other people
**18b**	(b) I am as healthy as anybody I know

**18c**	(c) I expect my health to get worse
**18d**	(d) My health is excellent
**General health scale with 18**	0.7481045
**General health scale with 18a**	0.68355
**General health scale with 18b**	0.73296
**General health scale with 18c**	0.70759
**General health scale with 18d**	0.83714
